# Cutoffs, sensitivity and specificity of the Ewing battery in evaluating autonomic nervous system disorders: a systematic review

**DOI:** 10.1007/s10286-025-01185-x

**Published:** 2026-01-20

**Authors:** Theodora R. Barkoula, Christiana Ioannou, Martina Rekatsina, Kassiani Theodoraki, Panagiotis Zis

**Affiliations:** 1 Medical School, University of Cyprus, Nicosia, Cyprus; 2 First Department of Anaesthesiology, Aretaieion Hospital, University of Athens School of Medicine, Vassilissis Sofias 76, 11528 Athens, Greece

**Keywords:** Ewing, Dysautonomia, Valsalva, Handgrip, Deep breathing, 30:15 ratio

## Abstract

**Purpose:**

Many methods have been developed for the assessment of dysautonomia, but they are often unreliable and not readily applicable to daily practice. Among the available investigation methods, the Ewing battery, which includes five non-invasive cardiovascular reflex tests, have become the reference standard in assessing dysautonomia and have been utilized for more than four decades. This systematic review evaluates the diagnostic thresholds and diagnostic performance of Ewing tests across studies in identifying autonomic nervous system (ANS) disorders.

**Methods:**

We conducted a comprehensive multi-database literature search, including PubMed, EMBASE, Web of Science, Scopus, and the Cochrane Library, using a pre-defined search strategy, for studies that applied the Ewing tests to assess autonomic dysfunction. We focused on studies that used continuous recordings and those reporting extractable numerical data, including either normative values or diagnostic cutoff thresholds for the Ewing tests. The Ewing parameters that were tested were Valsalva manoeuvre, heart rate variability during deep breathing (E/I ratio), the isometric handgrip, heart rate response to standing (30:15 ratio) and blood pressure response to standing.

**Results:**

Our analysis demonstrates good diagnostic performance of the Ewing tests, with sensitivity for individual components reaching up to 91% for Valsalva ratio (VR) in some cohorts. The normative data and respective cutoff points are influenced by age and sex. Optimal diagnostic performance was achieved when the Ewing battery was interpreted using the conventional criterion of ≥ 2/5 abnormal tests particularly when age-adjusted cutoffs were applied.

**Conclusion:**

The Ewing battery remains a highly effective tool for diagnosing ANS disorders, especially when age-adjusted normative thresholds are used. To further enhance its diagnostic performance, each laboratory should establish its own normative data reflective of the specific population it serves.

**Supplementary Information:**

The online version contains supplementary material available at 10.1007/s10286-025-01185-x.

## Introduction

Autonomic nervous system (ANS) disorders, as primary diseases or secondary complications of other diseases, are considered rare [1]. However, in daily practice, numerous clinical manifestations, in many cases atypical, may be due to dysautonomia, which can be associated with increased mortality and morbidity [1]. Symptoms such as orthostatic hypotension, tachycardia, reduced or loss of sweating ability, blurry vision, gastrointestinal symptoms and even cognitive changes are some of the manifestations of ANS dysfunction that are often underdiagnosed [1, 2]. As a result of the extensive distribution of the ANS, the exact mechanisms and structural changes that affect autonomic neuronal regulation in patients with dysautonomia remain obscure [1].

Several techniques have been proposed to study the ANS, but most methods either lack diagnostic value or require complex procedures or invasive methods [1]. Table 1 summarizes selected autonomic function tests adapted from Weimer [1], categorized by system tested (cardiovagal, adrenergic, sudomotor, investigational) [1]. The Ewing battery functions as a complete yet a simple non-invasive cardiovascular reflex testing system [3]. It contains assessments of the parasympathetic nervous system through heart rate variability response to Valsalva manoeuvre (Valsalva ratio, VR), heart rate response to standing (30:15 ratio) and heart rate response to deep breathing (E/I ratio) and assessments of sympathetic nervous system through blood pressure (BP) responses (BP response to standing and sustained handgrip). Table 2 provides a detailed description of the Ewing battery tests, as originally defined by Ewing and colleagues [2, 5, 15].

**Table 1:** Selected tests of autonomic function

Tests of autonomic function	Cardiovagal	Adrenergic	Sudomotor	Investigational
HR response to deep breathing	+			
HR response to the Valsalva manoeuvre	+			
HR response to standing (30:15 ratio)	+			
Diving reflex/cold face test	+			
HR response to cough	+			
HRV in rest	+			
Spectral analysis of HRV	+			
Transfer function analysis (non-linear dynamics)	+			
BP response to the Valsalva manoeuvre		+		
BP response to orthostatic stress		+		
BP response to standing		+		
Head up tilt test		+		
Sustained handgrip		+		
Squat test		+		
Plasma catecholamine levels (supine/standing)		+		
Microneurography		+		
Mental stress test		+		
Cold pressor test		+		
BP response to lying down/liquid metal/neck suction		+		
Spectral and transfer function of BP analysis		+		
Sympathetic skin response (SSR)			+	
Quantitative sudomotor axon reflex test (QSART)			+	
Thermoregulatory sweat test (TST)			+	
Silastic sweat imprint testing			+	
Pupillary testing (pharmacologic)				+
Pupillometry/pupillography				+
Urodynamics/cystometrogram with betanechol				+
Salivary testing/Schirmer test				+
Quantitive direct and indirect test of sudomotor function (QDIRT)				+
Cardiac fluorodopamine PET scanning				+
Penile plethysmography/papaverine injection				+

Source: Adapted from Weimer [1]

**Table 2:** Ewing cardiovascular reflex tests and definitions; composite criterion (≥ 2/5 abnormal) defines dysautonomia

Test	Physiological function	Brief procedure
Valsalva manoeuvre (VR)	Parasympathetic (cardiovagal)	Exhale against 40 mmHg for 15 s via mouthpiece; The ratio of the longest R–R interval shortly after the manoeuvre (within about 20 beats) to the shortest R–R interval during the manoeuvre is then measured. The result is expressed as the Valsalva ratio which is taken as the mean ratio from three successive Valsalva manoeuvres
Deep breathing (E/I ratio)	Parasympathetic (vagal)	The patient sits quietly and then breathes deeply and evenly at six breaths per minute (5 s in and 5 s out). The maximum and minimum heart rates during each 10-s breathing cycle are measured and the mean of the differences during three successive breathing cycles gives the ‘maximum-minimum heart rate’. An alternative way to express these changes is as a ratio of the heart rate at expiration to that at inspiration, the E/I ratio
Sustained isometric handgrip (ΔDBP)	Sympathetic (α-adrenergic)	To perform this test handgrip is maintained at 30% of the maximum voluntary contraction up to a maximum of 5 min, using a handgrip dynamometer, and the blood pressure measured each minute. The difference between the diastolic blood pressure just before release of handgrip and before starting is taken as the measure of response
HR response to standing (30:15 ratio)	Parasympathetic (baroreflex)	The subject is asked to lie quietly on a couch and then to stand up unaided. The heart rate response is expressed by the 30:15 ratio, which is the ratio of the longest R–R interval around the 30th beat after starting to stand up to the shortest R–R interval around the 15th beat
BP response to standing (ΔSBP)	Sympathetic adrenergic	This test is performed by measuring the blood pressure while the subject is lying down, and again 1 min after standing up. The difference in systolic blood pressure is taken as the measure of postural blood pressure change

Early cardiovascular autonomic neuropathy (CAN), 1 abnormal test; definite CAN, ≥ 2 abnormal; severe CAN, ≥ 2 abnormal plus orthostatic hypotension (adopted from Ewing et al. [2, 5])

The Ewing battery remains the most established and widely adopted clinical method for evaluating dysautonomia [3, 4, 11]; however, there are two major limitations in its application and interpretation. The first major limitation is in the interpretation of the results, because the normal values and the methodology vary between laboratories [4] and no universally accepted standardized protocol has been established. In their foundational publications, Ewing et al. [2, 5, 15] recommended the use of all five cardiovascular autonomic reflex tests as the diagnostic battery of autonomic neuropathy. They presented the procedure of each manoeuvre, physiological rationale, and diagnostic cutoffs but provided little guidance on patient’s preparation, optimum environment, sequence and rest intervals. Notably, Ryder and Hardisty [6] highlighted that while the Ewing battery is the reference standard in diagnosing dysautonomia, the variability in performing and duration of tests, and the lack of age-specific norms, may impact test sensitivity and specificity. Μethodological guidelines by O’Brien [7], Low [8, 9], Freeman et al. [10, 14], Spallone et al. [11, 12] and others [4, 13] expanded the procedural information in order to standardize the methodology of tests. They further proposed that a modified, simplified test battery could improve practicality and diagnostic accuracy [6].

The second limitation of tests is that they may pose an undue risk to the patient. Therefore, many studies and autonomic guidelines have excluded patients with atrial fibrillation (AF), permanent pacemaker or dependence on cardiac pacing, recent myocardial infarction or unstable cardiac disease from heart rate variability (HRV)-based testing [18, 19]. The Valsalva manoeuvre, handgrip, and other stress-based components of the Ewing battery may increase intrathoracic pressure and cardiac workload and are not recommended in patients with unstable ischemic heart disease or recent myocardial infarction (< 6 weeks) [19]. Severe respiratory conditions e.g. exacerbated chronic obstructive pulmonary disease (COPD) or asthma, are also a limitation in performing Ewing tests. The Valsalva manoeuvre and deep breathing require forceful respiratory efforts. In patients with impaired pulmonary function, these manoeuvres can provoke bronchospasm or hypoxia and should not be performed during acute decompensation [18]. Active retinal disease (e.g. recent retinal hemorrhage or proliferative diabetic retinopathy) is also a contraindication in performing Ewing tests because of transient increase of intraocular pressure, during handgrip test and Valsalva manoeuvre, which may exacerbate retinal pathology and precipitate hemorrhage in vulnerable patients [12, 21]. Moreover, the Ewing battery requires active and precise patient participation. In individuals unable to understand or comply with breathing rhythms or positional changes (e.g. advanced dementia), the tests are not feasible and the results are unreliable [18].

The establishment of validated population-adjusted cutoffs and standardized methodology leads to better clinical consistency and research reproducibility and improves patient outcomes by minimizing both underdiagnosis and false positives.

The purpose of this systematic review is to comprehensively evaluate and compare the diagnostic thresholds, sensitivity, and specificity of the individual tests comprising the Ewing battery across published studies. Specifically, we aim to synthesize evidence from high-quality studies that have applied the Ewing battery using standardized or well-described methodologies, in order to identify which cutoff values demonstrate the most robust diagnostic performance for detecting autonomic dysfunction. In addition, this review seeks to highlight methodological variability and gaps in the existing literature, identify key areas requiring further investigation, and clarify unresolved questions regarding test interpretation and optimization. Ultimately, we intend for this systematic review to serve as a practical, evidence-based reference for clinical and research laboratories that currently offer, or are considering the implementation of, autonomic function testing using the Ewing battery.

## Methods

### Literature search strategy

An online literature search was conducted across five databases (PubMed, EMBASE, Scopus, Web of Science, and the Cochrane Library) on November 18, 2025 using two medical subject heading (MeSH)-based search strings: (a) “Ewing” in the title or abstract; (b) “autonomic OR dysautonomia OR Valsalva OR deep breathing OR handgrip OR standing” in the title or abstract.

Three filters were applied; these ensured that the results were available in English, involved only human participants, and provided access to the full text. As the “full text available” filter was not supported across all databases, full-text accessibility for non-PubMed records was assessed manually during the eligibility phase.

Articles were eligible regardless of whether the full text was open access or subscription-based. All peer-reviewed full-text manuscripts obtainable through publisher platforms, institutional subscriptions, or interlibrary resources were included.

The reference lists of all eligible full-text articles were screened manually to identify any additional relevant studies. No other manual search techniques (such as forward citation tracking, screening of reviews, or expert recommendations) were used.

### Inclusion and exclusion criteria

Articles deemed eligible to be included in this review had the following characteristics:

- The studies applied one or more Ewing battery tests for autonomic function assessment.
- The studies performed continuous digital recording of signals.
- The study subjects were human.
- The study language was English.
- The studies were available in full text.

Articles were excluded for the following reasons:

- Studies were not related to dysautonomia.
- Studies did not provide extractable numerical data relevant to Ewing test interpretation, defined as one or more of the following:
  1. Normative values in healthy/reference groups (means, standard deviations, percentiles, or confidence intervals).
  2. Explicit cutoff values for abnormal responses.
  3. Diagnostic accuracy metrics derived from Ewing tests (e.g. sensitivity, specificity). Where applicable, the Youden index was calculated to provide a balanced interpretation of diagnostic performance. The Youden index is defined as Sensitivity + Specificity − 1 and ranges from 0 to 1, with higher values indicating greater overall discriminatory ability [16].
- Article was a review, letter to the editor, single case report, conference presentations.
- Case series with ≤ 10 participants in order to ensure adequate methodological robustness, as very small samples lack statistical stability, provide insufficient distributional information for deriving or interpreting cutoff values, and do not allow meaningful assessment of diagnostic performance.
- The risk of bias was considered to be high based on the risk of bias assessment described in detail below.

Three researchers independently screened all search results. Titles and abstracts were assessed during the primary screening phase to identify records eligible for full-text evaluation. Full texts were then examined in a secondary screening to determine eligibility for inclusion in the review. Any discrepancies were resolved by consensus.

### Risk of bias assessment

Risk of bias was assessed independently by two reviewers using the appropriate Joanna Briggs Institute (JBI) critical appraisal tool for each study design (1 randomized controlled trial, 2 cohort studies, 7 case–control studies, and 41 analytical cross-sectional studies) [20]. Each study was evaluated across domains including participant selection, measurement validity and reliability, identification and management of confounding, and appropriateness of statistical analysis, with discrepancies resolved by consensus. As JBI does not recommend numerical scoring, author-defined, percentage-based cutoffs—an approach widely used in systematic reviews—were applied to categorize studies as low, moderate, or high risk of bias based on the total score (for randomized controlled trials—low, ≥ 10/13; moderate, 7–9/13; high, ≤ 6/13; cohort studies—low, ≥ 8/11; moderate, 6–7/11; high, ≤ 5/11; case–control studies—low, ≥ 7/10; moderate, 5–6/10; high, ≤ 4/10; analytical cross-sectional studies—low, ≥ 6/8; moderate, 4–5/8; high, ≤ 3/8).

### Ethical approval

This article is based on previously conducted studies and does not contain any new studies with human participants or animals performed by any of the authors. Therefore, no approval from the local or national ethical committee was requested.

### Project registration

The screening process was carried out in accordance with the Preferred Reporting Items for Systematic reviews and Meta-Analyses (PRISMA) guidelines [22]. The PRISMA 2020 checklist is available as Supplementary material.

This project was registered in the PROSPERO database for meta-analysis and systematic reviews (Registration number CRD42024606865). No amendments were made to the registered protocol.

## Results

### Study selection

The literature search strategy identified a total of 1694 records across all platforms. Application of the English-language filter reduced the number to 1471 records. Limiting the results to human studies further narrowed the set to 1324 records. Finally, applying the full-text availability filter resulted in 1296 records eligible for screening.

**Fig. 1:**
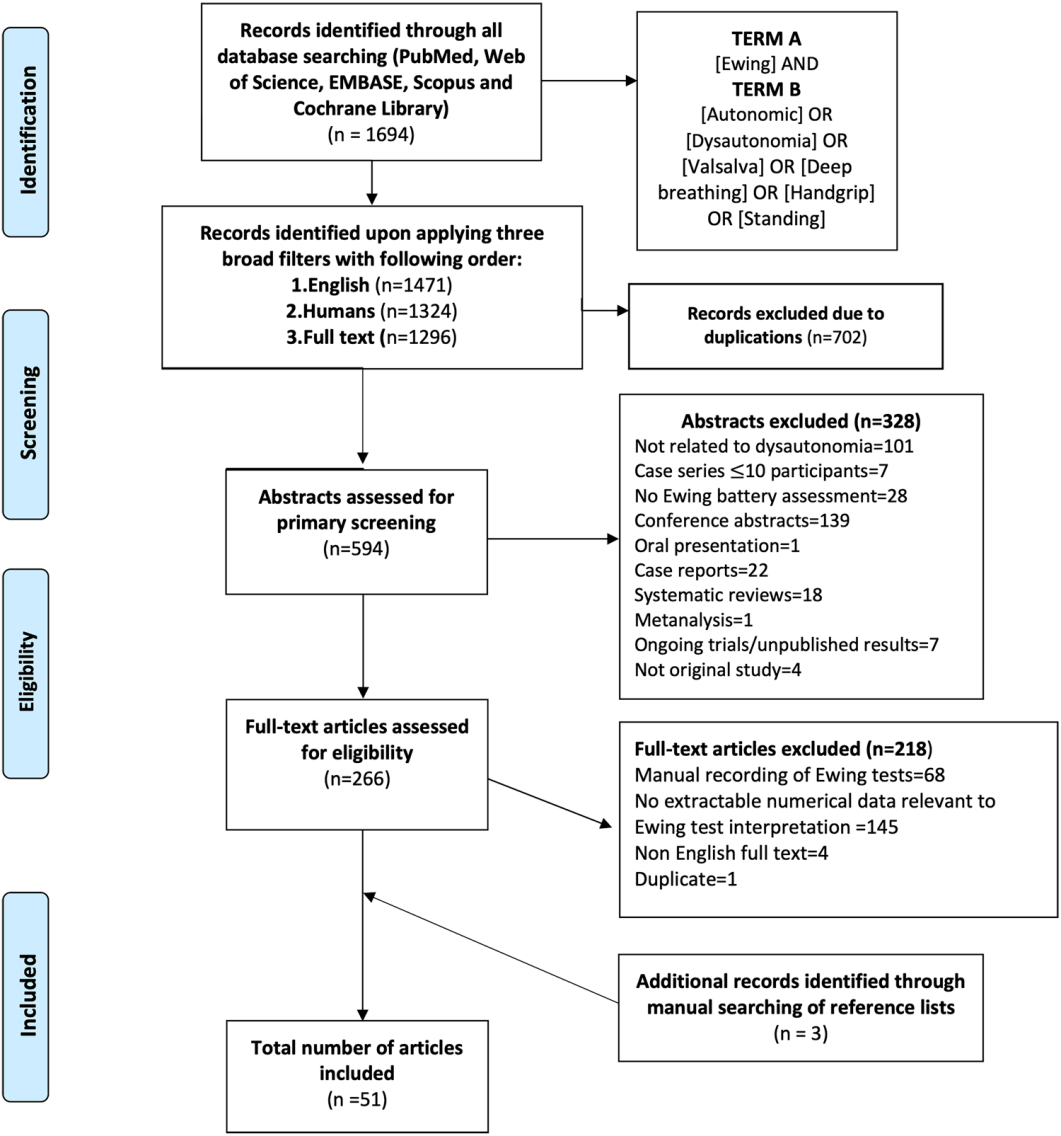
PRISMA diagram

Following the removal of 702 duplicates, 594 unique records underwent abstract screening where 328 were excluded. The remaining 266 underwent full-text screening where 218 were excluded. A total of 48 studies met the inclusion criteria, and three additional articles were identified through manual reference screening, resulting in 51 studies included in the present review. Figure 1 summarizes in detail the study selection process.

Risk of bias assessment showed that 24 studies were judged to be at low risk of bias, whereas 27 studies demonstrated moderate risk of bias, according to the relevant JBI appraisal criteria. As such, no study was excluded on the basis of methodological quality alone.

### Methodological reporting patterns across studies

Across the 51 included studies, 30 performed the complete Ewing battery (all five tests), 14 performed four tests, and 7 performed three tests. At the individual test level, 49 performed the Valsalva ratio, 50 assessed the deep breathing heart rate variability (E/I ratio), and 33 studies evaluated the sustained isometric handgrip test (ΔDBP), 48 studies assessed the heart rate response to standing (30:15 ratio) and 47 evaluated the orthostatic systolic blood pressure fall (ΔSBP).

As emphasized by Zygmunt and Stańczyk [17], parasympathetic manoeuvres (deep breathing, 30:15 ratio, Valsalva HR response) recover within seconds, whereas sympathetic adrenergic adjustments in vascular tone have a slower onset and resolution [2, 17, 26]. In our review, however, only five of the included studies explicitly documented the sequence of tests [15, 23–26], underscoring a methodological limitation that may contribute to variability across published results.

A baseline control period (i.e. remaining in a supine position for a specific period of time) before testing was described in 35 studies, while 16 gave no baseline control information. Four studies [26, 27, 42, 43, 60] documented baseline adjustments and two studies specifically mentioned a 1-h baseline recording for adjustment at rest prior to testing, to ensure signal stability and calibration accuracy [42, 43, 68]. These observations highlight how rarely formal baseline standardization is operationalized. General procedure details were provided in 42 studies and environment/laboratory conditions were described in 38 studies, yet the exact rest intervals between tests and precise baseline computation windows were seldom stated. Overall, while many authors acknowledge pre-test “rest” and laboratory conditions, the sequence of tests, rest-interval durations, and explicit baseline definition are inconsistently reported, which creates unavoidable variability and limits reproducibility in clinical practice.

### Normative values applied across studies

Among the studies analyzed, the five standard Ewing tests presented heterogeneous cutoff definitions, although the classical cutoffs described by Ewing remain the most widely applied.

### Valsalva ratio

The Valsalva ratio (VR) cutoff values established across 49 studies showed a uniform decrease with age. The most commonly used cutoffs were the standard Ewing reference values [15], that were use in 37 studies [15, 23–25, 28, 38, 42, 43, 46, 50–54, 59–62, 66–70, 73–75, 85–96]. 12 studies applied age-adjusted lower limits of normal [26, 27, 29–31, 36, 37, 41, 44, 45, 47, 76]. Keller et al. [37] and Pavlovic et al. [41] used normal VR thresholds adjusted for age based on the initial findings of Levin who established 1.50 as the lower limit of normal for a large cohort of 200 healthy subjects and observed that 96% of participants had values above 1.50 [40]. Montesano et al. [30] applied O’Brien’s age-adjusted reference values [7] in a cohort of 25 young patients with obstructive sleep apnea syndrome (OSAS) and 25 age-matched controls. The observed values demonstrate a small reduction in parasympathetic tone, which matches previous findings showing VR values decreased with age [34, 35, 40, 43], and that higher VR cutoffs in younger adults represent the strong vagal modulation that occurs during early adulthood [34, 40, 43]. The best performance for the Valsalva ratio was reported by Lin et al. [31] in a study of 90 patients with diabetes and 20 controls, with a sensitivity of 90% and a specificity of 95%, yielding a Youden index of 0.85; the authors applied the cutoff values recommended by Spallone et al. [12], as shown in Table 3. No studies implemented sex-specific diagnostic thresholds [44, 45]. Some female-dominant cohorts showed values that concentrated at the higher end of the normative window [37, 46, 47], which matches previous physiological research indicating that women experience stronger parasympathetic rebound [9, 48, 49]. The observed differences, though, between sexes have not, on the basis of current literature, led to the establishment of separate diagnostic cutoffs for men and women.

**Table 3:** Cutoff values for cardiovascular autonomic reflex tests: reported ranges and proposed thresholds for laboratories not having own normative data (Ewing, age-specific, and review-proposed values)

Test	Ewing proposed cutoffs [15]	Children	≤ 40 years^a^	41–60 years^a^	61–70 years^a^	≥ 70 years^a^	Review proposed cutoffs		Reason
Valsalva ratio (VR)^b^	≥ 1.21	≥ 1.35 [30]	≥ 1.21 [15] to ≥ 1.35 [12]	≥ 1.21 [15] to ≥ 1.24 [12]	≥ 1.19 [12] to ≥ 1.21 [15]	≥ 1.15 [12] to ≥ 1.21 [15]	Age group (years)	Value	Selected based on Lin et al. (90 patients/20 controls) which provided the highest sensitivity (90%) and the highest Youden index (0.85)
							≤ 29	≥ 1.42	
							30–39	≥ 1.35	
							40–49	≥ 1.29	
							50–60	≥ 1.24	
							61–70	≥ 1.19	
							71–80	≥ 1.15	
							[12]		
E/I ratio	NR	≥ 1.17 [30]^c^	≥ 1.10 [7] to ≥ 1.19 [12]	≥ 1.04 [7] to 1.11 [12]	≥ 1.02 [7]– ≥ 1.08[12]	≥ 1.00 [7] to ≥ 1.05 [12]	Age group (years)	Value	Selected based on Lin et al. (90 patients/20 controls) which provided the highest sensitivity (80%) and the highest Youden index (0.75)
							≤ 29	≥ 1.24	
							30–39	≥ 1.19	
							40–49	≥ 1.15	
							50–60	≥ 1.11	
							61–70	≥ 1.08	
							71–80	≥ 1.05	
							[12]		
E/I bpm difference (HR variation max–min)	≥ 15 bpm		≥ 7 bpm [7] to ≥ 15 bpm [15]	≥ 4 bpm [7] to ≥ 15 bpm [15]	≥ 3 bpm [7] to ≥ 15 bpm [15]	≥ 2 bpm [7] to ≥ 15 bpm [15]	≥ 15 bpm [15]		Selected based on Viswanathan et al. (50 patients/20 controls) which provided the highest sensitivity (84%) and the highest Youden index (0.84)
Isometric handgrip	≥ 16 mmHg		Ν/Α	Ν/Α	Ν/Α	Ν/Α	≥ 16 mmHg [15]		Selected based on Baschieri et al. (99 patients/38 controls) which provided the highest sensitivity (85%) and the highest Youden index (0.79), adopted Ewing thresholds
30:15 ratio	≥ 1.04	≥ 1.20 [30]	≥ 1.04 [15] to ≥ 1.12 [7]	≥ 1.04 [15] to ≥ 1.12 [7, 12]	≥ 1.02 [7, 12] to ≥ 1.04 [15]	≥ 1.00 [12] to ≥ 1.04 [15]	Age group (years)	Value	Cardone et al. (235 patients/224 controls) provided the strongest diagnostic accuracy for the 30:15 ratio. Age-related reference limits applied here follow Spallone’s recommendations, which integrate the broader normative work of the Cardone group
							≤ 29	≥ 1.15	
							30–39	≥ 1.11	
							40–49	≥ 1.07	
							50–60	≥ 1.05	
							61–70	≥ 1.02	
							71–80	≥ 1.00	
							[12]		
ΔSBP (systolic drop)	≤ 30 mmHg		Νo age-specific cutoffs have been reported. Studies use either ≤ 20 mmHg or ≤ 30 mmHg as cutoff				≤ 30 mmHg [15]		Selected based on Lin et al. (90 patients/20 controls) which provided the highest specificity(100%) and the highest Youden index (0.4)
ΔDBP (diastolic drop)			Νo age-specific cutoffs have been reported. Most studies use ≤ 10 mmHg as cut off				Νo study described normative data		

*E/I* exhale/inhale, *NR* not reported, *bpm* beats per minute, *HR* heart rate, *ΔSBP* systolic blood pressure drop, *ΔDBP* diastolic blood pressure drop

^a^Normative values in controls across 51 studies

^b^The study from Levin et al. [40] uses a uniform cutoff of 1.50 for VR

^c^On the basis of the Spallone et al. study [12] it can be argued that the cutoff of ≥ 1.24 which applies to people ≤ 29 is applicable to children too

### Heart rate response to deep breathing (E/I ratio)

Across the included studies, 44 studies applied the classical Ewing diagnostic thresholds [15, 24, 25, 27–29, 32, 37–39, 41–46, 50, 51, 53, 54, 59–62, 66–70, 72–76, 85–95] whereas 6 studies [23, 26, 30, 31, 36, 47] used age-adjusted lower limits of normal derived from the normative datasets of O’Brien et al. [7] and the recommendations of Spallone et al. [12].

E/I ratio values showed a clear age-related decline, ranging from up to 1.17 in young adults [24, 31, 32, 50] to 1.02 in those over 75 years [23, 31, 32] which confirms previous findings about age-related vagal withdrawal [5, 12, 34, 43]. The influence of age on heart rate response to deep breathing was originally reported by Levin et al. in a physiological study of respiratory sinus arrhythmia [40].

Our review findings are consistent with previous literature which indicates that parasympathetic tone diminishes over the lifespan, in part as a result of declining baroreflex sensitivity and reduced cardiac compliance [34, 35]. The best diagnostic accuracy was reported by Viswanathan et al. [53], in 50 patients with diabetes and 20 healthy controls, who applied the standard Ewing reference values [15] and Lin et al. [31], in 90 patients with diabetes and 20 controls, who applied the age-adjusted normal values based on the recommendations for the use of cardiovascular tests by Spallone et al. [12]. Although Ewing et al. [15] reported a clear sex difference in the deep-breathing ΔHR (31 ± 9 bpm overall; men 34 ± 10 vs. women 25 ± 8), for the E/I ratio, no consistent sex-based variation was evident across studies. Most studies used the same normal range, without reporting separate cutoffs by sex [32, 38, 51, 54]. Published studies have reported inconsistent findings regarding sex differences in tonic vagal modulation of HR. Some have found a significant difference between men and women [9, 55, 56], whereas others have suggested that the differences exist but are too small to justify separate cutoffs [39, 57].

### Isometric handgrip

The isometric handgrip (ΔDBP) test showed low variability regardless of age and sex. All studies included in this review used the ΔDBP cutoff ≥ 16 mmHg during the sustained handgrip test without establishing age- or sex-specific thresholds [32, 36, 45, 62, 66]. Ewing et al. [15] presented normative data from 139 healthy controls in the age range 16–69 years. Men in this age category showed larger pressor response than women (34 ± 10 mmHg vs. 25 ± 8 mmHg). Correlation analysis showed no significant association between ΔDBP and age in men aged 17–64 years (*r* = 0.02, NS), whereas women aged 12–52 years demonstrated a moderate, statistically significant age-related decline (*r* = − 0.35, *p* < 0.05). Ewing’s data demonstrated that in women aged 12–52 years there was a moderate, statistically significant age-related decline in ΔDBP [15], and a uniform threshold may overestimate sympathetic dysfunction in older women. The optimum diagnostic performance was found in the study by Baschieri et al. [27] (Table 3).

### Heart-rate response to standing (30:15 ratio)

Regarding the 30:15 ratio, 43 studies [15, 24, 25, 28, 29, 37–39, 41–46, 50, 51, 53, 54, 59–62, 66, 67, 69, 70, 72–76, 85–96] used a normal threshold of ≥ 1.04, consistent with Ewing’s original study that applied a single uniform cutoff value of 1.04 for all age groups [15]. Five studies categorized subjects into age groups [30–32, 36, 47].

Age-stratified cutoff trends were evident in all parasympathetic tests. The reported values ranged significantly, with younger adults showing normal values up to 1.90, and older adults of the age group 70–75 years old, declining toward 1.02 [31, 32]. These findings are consistent with previous studies, which show how cardiovagal tone and baroreflex sensitivity decrease with age [9, 34, 35]. It is worth highlighting the study by Cardone et al. [36], the largest study conducted in 224 healthy individuals, using the most robust methodology, which demonstrated a clear age effect on the 30:15 ratio through regression analysis, and applied lower limit of normal (LLN) thresholds defined at the 5th percentile (P5), with the first percentile (P1) denoting abnormality [12], as shown in Table 3. For the 30:15 ratio, both male- and female-representative studies consistently used universal cutoffs for both sexes [29, 31, 32, 37, 38].

### Blood pressure response to standing

Despite the well-documented increase in orthostatic BP drop (ΔSBP) with age [9, 43, 58], the reviewed 47 studies applied stable cutoffs across all age groups. For ΔSBP, two cutoff thresholds for abnormality were used across the literature: ≥ 30 mmHg in 38 studies [15, 23, 29–32, 36, 37, 39, 41–43, 45–47, 50, 53, 59–62, 66, 68–70, 72–76, 85, 87, 90–96], and ≥ 20 mmHg in 9 studies [24, 28, 38, 44, 51, 54, 67, 86, 89], reflecting a binary distribution rather than a continuous range. Importantly, the original normative distributions by Ewing et al. [15] showed orthostatic SBP changes from − 30 to + 15 mmHg in healthy controls, with very few falls ≥ 20 mmHg and none > 30 mmHg, supporting the higher (≥ 30 mmHg) threshold for specificity. The optimum diagnostic performance was found by Lin et al. [31], who applied the cutoff of ≥ 30 mmHg (adopted from Spallone recommendations [12]), measured at both the first and the second minute after standing. These results are consistent with the original findings of Ewing and colleagues, who first defined a systolic blood pressure fall ≥ 30 mmHg within 2 min of standing as abnormal [15]. According to Spallone’s recommendations for the use of cardiovascular tests [12], although some investigators have suggested that a diastolic rather than a systolic drop may better characterize autonomic failure, this interpretation is problematic. In patients with diabetes, SBP declines are detected more often than DBP ones [34]. Moreover, the absolute diastolic change after standing is typically smaller and therefore more prone to measurement variability when using a standard sphygmomanometer [12]. As emphasized by Spallone et al. [12], these issues reinforce the preference for systolic thresholds in both clinical practice and research. Subsequent literature has demonstrated that the magnitude of orthostatic BP decline is influenced by age, with older individuals exhibiting a greater physiological drop, due to baroreflex and vascular changes [9, 35, 58]. A universal cutoff may overestimate dysfunction in the young and underestimate it in the elderly [10, 58]. When supine BP is ≥ 160 mmHg or ≤ 120 mmHg, orthostatic ΔSBP is confounded—high baseline BP exaggerates the fall (false positives), while low baseline BP attenuates it (false negatives) [12]. Studies such as Finucane et al. [58] and Freeman et al. [10] support the use of age-adjusted thresholds for improved diagnostic accuracy. In particular, Finucane et al. [58] demonstrated age- and sex-related changes in blood pressure during orthostatic challenge 60 s after standing, in a cohort of 4475 community-dwelling adults, who underwent beat-to-beat recordings of Ewing battery tests. The Toronto Consensus [11] on cardiovascular autonomic neuropathy (CAN) in diabetes recommends adopting the Ewing criterion of a systolic blood pressure fall ≥ 30 mmHg at 1 and 2 min of standing as the abnormal cutoff. The consensus definitions of orthostatic hypotension, endorsed by the American Autonomic Society and the American Academy of Neurology [10], recommend a threshold of ≥ 20 mmHg for ΔSBP or ≥ 10 mmHg for ΔDBP within 3 min of standing. The higher systolic threshold, allying with Ewing’s classical cutoffs [15], enhances specificity for diagnosing CAN [15, 58], whereas the broader orthostatic hypotension definition applies more generally to clinical syndromes of impaired orthostatic BP regulation [10]. In the TILDA normative study [58], systolic falls ≥ 30 mmHg were observed only beyond the 95th percentile of healthy controls across all age groups, supporting their use as a highly specific threshold for autonomic failure. With regard to sex-related differences, no study of our review provided distinct cutoff values for male and female subjects. For ΔSBP, some studies applied a uniform threshold of ≥ 30 mmHg, while others used ≥ 20 mmHg, regardless of sex [37, 46, 54, 61, 62]. Physiology research shows that men tend to show greater SBP variability when changing positions because of their higher sympathetic tone and more muscle mass, and women, especially those premenopausal, tend to have reduced responses because of hormonal influences such as oestrogen’s vasodilatory effects [9, 58, 64, 65]. However, the known sex-related differences in baroreflex and autonomic responses have not led to the establishment of separate cutoffs for men and women in current clinical practice [9, 34, 63].

Table 3 presents the Ewing-proposed cutoffs, age-specific reference values and our review’s proposed diagnostic thresholds based on the best diagnostic performance.

### Classification criteria across studies

In order to evaluate diagnostic accuracy and test performance of the Ewing battery it is essential to consider how autonomic dysfunction was diagnosed across studies.

Our systematic review of 51 studies revealed substantial heterogeneity in diagnostic criteria. The most frequently applied definition (31 studies) was the classical Ewing criteria, which consider dysautonomia present if two or more out of five standardized autonomic tests are abnormal [15]. This method, originally described by Ewing and Clarke [15], remains the most popular model used in clinical and epidemiological studies. Ten studies [27, 36, 38, 41, 62, 67, 73, 75, 76, 87] employed a cumulative scoring model, typically scoring each examination with 0 (normal), 1 (borderline), or 2 (abnormal), and providing a diagnostic cutoff score of ≥ 4 out of 10 points. This approach is particularly relevant in patients with mixed results or borderline impairment and is especially useful when borderline findings are common [11]. One study employed a similar semi-quantitative categorical point system with an intermediate (0.5) value for borderline responses [67]. The reliability and consistency of such systems in detecting subclinical or early-stage cases remain uncertain. Six studies utilized stricter definitions, with ≥ 2 abnormal heart rate (HR)-based tests or a combination of parasympathetic and sympathetic dysfunction [26, 28–30, 32, 86]. This approach is consistent with previous literature [34, 71], where the diagnostic significance of parasympathetic degradation as a marker of early autonomic failure was emphasized. Interestingly, one study employed a percentile cutoff approach, classifying test results as abnormal if they were less than the 5th percentile of control distributions [61]. This method is data driven and becomes more prevalent in population-level screening studies [34]. Although such percentile-based thresholds improve population-specific diagnostic precision, they complicate direct comparisons with studies employing traditional Ewing-defined criteria. Two studies did not provide sufficient information regarding the diagnostic reference standard. Overall, while the Ewing criteria of ≥ 2/5 tests being abnormal remain dominant, the increasing use of scoring systems and statistical thresholds reflects a broader effort to increase sensitivity and tailor diagnosis to specific populations. Semi-quantitative score systems have been applied by Ewing and co-workers [15], and other studies in order to identify progression or improvement of autonomic dysfunction, as well as for monitoring treatment response [46, 52, 66]. Methodological non-uniformity continues to represent a key barrier to standardization, meta-analysis, and the creation of universally applicable guidelines. Since the applied diagnostic standard directly determines the diagnostic accuracy, it should be explicitly stated and justified in all studies.

### Diagnostic accuracy of Ewing battery

As reported previously, across our review studies, different diagnostic reference standards have been applied, most frequently the criterion of ≥ 2/5 abnormal cardiovascular reflex tests. This definition derives from the original work of Ewing and Clarke [15] and was subsequently endorsed by consensus statements [12]. However, no study has validated these thresholds against an independent reference standard of autonomic neuropathy, rendering them essentially operational and to some extent arbitrary. Although normative data exist for individual test values in healthy populations, no investigation has formally demonstrated that the composite cutoff (e.g. ≥ 2/5 abnormal tests) provides optimal discrimination between healthy and diseased groups.

Nevertheless, evidence from the present review supports the specificity of this definition. In 35 studies that reported the distribution of Ewing battery results in healthy controls, the vast majority of subjects were classified as 0/5, with only occasional cases presenting with 1/5 abnormal tests (borderline findings). Specifically, 14 studies explicitly stated that no healthy controls presented with ≥ 2/5 abnormalities, confirming the specificity of this criterion [25–27, 29, 32, 37, 39, 46, 47, 50, 53, 62, 68, 76]. In a smaller number of reports, isolated cases with ≥ 1/5 abnormal tests were noted but without evidence of multiple failures [15, 36, 61, 75]. Taken together, these observations demonstrate that ≥ 2 abnormal Ewing tests are virtually absent in healthy populations, thereby reinforcing the use of this cutoff as the reference standard definition of definite CAN in line with Ewing’s original proposal [15] and the Spallone/Toronto recommendations [11, 12].

### Valsalva ratio

The Valsalva ratio had sensitivity up to 91% and specificity up to 100%, depending on the applied reference standard [27, 31, 36]. The best performance was observed in a study that include 90 patients with diabetes and 20 controls by Lin et al., who showed a Youden index of 0.85, using a semi-quantitative scoring system as a reference standard—0 for normal, 0.5 for borderline, and 1 for abnormal responses—and classification as CAN if the sum of the score was ≥ 2 points [31], as indicated in Table 4. This approach includes the inclusion of borderline values in the scoring, potentially identifying milder presentation of parasympathetic dysfunction. In that cohort, age-adjusted cutoffs were applied, according to the recommendations of Spallone [12]. A similar performance was indicated by Baschieri et al. [27], where the Valsalva ratio reached a Youden index of 0.83 using the ≥ 4/10 Ewing score as the diagnostic cut-point (Table 4). Unlike other studies, the diagnostic reference standard was not an Ewing score threshold, but the final clinical diagnosis of multiple system atrophy (MSA) versus Parkinson’s disease (PD) confirmed after longitudinal follow-up, against which the cardiovascular reflex tests were evaluated. The study included 34 patients with MSA, 65 patients with PD without overt dysautonomia, and 38 healthy controls, and applied the classical Ewing cutoffs. Overall, studies that utilized age-adjusted graded scores reported improved diagnostic performance, which underlines the importance of methodological accuracy in optimizing the discriminative capacity of the test. Studies with age-adjusted VR cutoffs demonstrated greater consistency of performance across age groups, suggesting the advantage in applying normative, age-stratified cutoffs to achieve optimal diagnostic accuracy.

**Table 4:** Diagnostic performance metrics (sensitivity, specificity, Youden index) of Ewing cardiovascular autonomic tests, derived from the studies synthesized in this review

VR (Sens/Spec/Youden)	E/I (Sens/Spec/Youden)	HG (Sens/Spec/Youden)	30:15 (Sens/Spec/Youden)	ΔSBP (Sens/Spec/Youden)	Gold standard	Healthy (*n*)	Patients (*n*)	Study
42.7 / 93.0 / 0.36	44.0 / 77.1 / 0.21	69.3 / 57.6 / 0.27	8.0 / 83.3 / 0.09	19.0 / 95.0 / 0.14	≥ 4/10	55	75	[67]
10.0 / 100 / 0.10	60.0 / 100 / 0.60	26.7 / 93.3 / 0.20	0.0 / 100 / 0.00	–	parasympathetic score ≥ 4/6 or sympathetic ≥ 1/4	30	30	[28]
42.9 / 95.2 / 0.38	52.4 / 76.2 / 0.29	57.1 / 61.9 / 0.19	33.3 / 85.7 / 0.19	4.8 / 100 / 0.05	≥ 4/10	21	21	[75]
90 / 95 / 0.85*	80 / 95 / 0.75	27.5 / 100 / 0.27	52.5 / 95 / 0.48	40 / 100 / 0.40*	≥ 2/5	20	90	[31]
22.7 / 100 / 0.23	81.8 / 100 / 0.82	60.8 / 100 / 0.61	17.4 / 100 / 0.17	26.1 / 100 / 0.26	≥ 4/10	40	36	[73]
–	50 / 96 / 0.46	14.8 / 100 / 0.15	42.3 / 99 / 0.41	24.6 / 100 / 0.25	Parasympathetic: ≥ 2/3 HR-based tests abnormal; Sympathetic: ≥ 1/2 BP-based tests abnormal	100	142	[32]
16.67 / 96 / 0.13	22.2 / 96 / 0.18	–	22.2 / 96 / 0.18	–	Parasympathetic: ≥ 2/3 HR-based tests abnormal; Sympathetic: ≥ 1/2 BP-based tests abnormal;Combined: ≥ 2 HR + ≥ 1 BP based tests abnormal	25	25	[30]
–	–	70 / 75 / 0.45	–	–	≥ 4/10	20	20	[76]
25 / 89.7 / 0.15	26.9 / 94.9 / 0.22	–	76.5 / 57.9 / 0.34	15.7 / 94.9 / 0.11	≥ 2/5	41	90	[74]
91 / 92 / 0.83	88 / 83 / 0.71	85 / 94 / 0.79*	–	–	≥ 4/10	38	99	[27]
44 / 100 / 0.44	84 / 100 / 0.84*	44 / 100 / 0.44	60 / 100 / 0.60	8 / 100 / 0.08	≥ 2/5	20	50	[53]
46.7 / 94.4 / 0.41	64 / 93.1 / 0.57	–	77.3 / 90.2 / 0.68*	–	≥ 4/10	224	235	[36]
0 / 100 / 0	46.2 / 84.6 / 0.31	23.1 / 92.3 / 0.15	15.4 / 100 / 0.15	0 / 100 / 0	≥ 2/5	13	13	[42]

*Asterisks highlight the best diagnostic performance metrics for each test

### Heart rate response to deep breathing (E/I ratio)

Heart rate response to deep breathing had the most balanced diagnostic accuracy, reaching up to 88% sensitivity and up to 100% specificity [27, 28, 31, 36, 53, 73]. Viswanathan et al. achieved a Youden index of 0.84 with the reference standard of ≥ 2/5 abnormal Ewing tests and demonstrates the most balanced combination of sensitivity and specificity among the included studies, with 29 of 50 patients with diabetes classified as having definite dysautonomia compared to none of the 20 healthy controls [53] (Table 4). It is worth highlighting that the study by Cardone et al. [36] reported an E/I ratio sensitivity of 64% and specificity of 93% (Youden index = 0.57) using the ≥ 4/10 Ewing score as the reference standard (Table 4). The study employed a rigorous methodological approach with well-defined scoring criteria and cutoffs, and was strengthened by the inclusion of a large number of participants with normal autonomic function (*n* = 224), alongside 75 patients with diabetes and definite dysautonomia and 160 without dysautonomia, which reinforces the specificity estimate and reduces spectrum bias.

### Sustained handgrip

The sustained handgrip (HG) test showed sensitivity up to 85% and specificity up to 100% [27, 31, 32, 53, 73, 76]. The best Youden index of 0.79 was reported by Baschieri et al. where the reference standard was the ≥ 4/10 with a 0–10 scoring system and the classical Ewing cutoffs were applied, as previously reported [27] (Table 4). In all studies, it maintained stable diagnostic value of ≥ 16 mmHg across ages. Performance was stronger in younger to middle-aged populations [27, 76]. As with the BP response to standing test, the HG demonstrated modest sensitivity, making its primary value confirmatory rather than a tool for detecting early ANS disorder [27, 31, 73, 76]. While overall performance was generally lower than that of parasympathetic indices, these findings support its role in the diagnostic work-up.

### Heart-rate response to standing (30:15 ratio)

The 30:15 ratio exhibits sensitivity scores up to 77% with specificity as high as up to 100% [31, 32, 36, 53]. The highest Youden index of 0.68 occurred in the study by Cardone et al., who, as previously noted, used the diagnostic criterion of ≥ 4/10 Ewing score [36]. This study provided the strongest confirmed evidence in all age groups (Table 4). Since the test has been reported to be highly age-related—with lower diagnostic yield in the elderly due to decreased autonomic reflex function—the application of age-corrected cutoffs in the Cardone study likely contributed to its consistently balanced diagnostic performance.

### Blood pressure response to standing

The blood pressure (BP) response to standing test had low sensitivity as a single test reaching up to 40% but a high specificity uniformly greater than 94%, justifying its application in the confirmation of proven autonomic dysfunction but not for early detection [31, 32, 53, 67, 74]. The highest Youden index was 0.40 indicating low discriminative ability compared to parasympathetic testing and was reported by Lin et al. with the diagnostic reference standard criterion of semi-quantitative point system, as previously reported, who applied for ΔSBP the cutoff of ≥ 30 mmHg, measured at both the first and the second minute after standing [31] (Table 4). This finding highlights that even with a robust reference standard, ΔSBP is highly specific but not sensitive enough, limiting its independent use in detecting dysautonomia.

## Discussion

The present review has the following key findings:

1. There is a strong age-dependence of parasympathetic indices consistent with contemporary guidance [78]. Across the 51 reviewed studies, the E/I ratio, 30:15 ratio, and Valsalva ratio all showed a progressive decline in normal values with advancing age. These trends are consistent with reduced cardiovagal tone and baroreflex sensitivity in ageing and highlight the importance of applying age-adjusted thresholds for accurate diagnosis. The original landmark works by Ewing and Clarke [15] and Cardone et al. [36] demonstrated key physiological characteristics of cardiovascular reflexes, including the age-related performance effects. The studies by Lin et al. [31] and Baschieri et al. [27] represent methodologically robust, high-quality studies that can serve as anchor points for both clinical application and further research.
2. There is minimal age-adjustment for sympathetic indices despite known physiological changes [15]. ΔSBP and ΔDBP thresholds were largely applied uniformly across ages, even though literature shows that orthostatic SBP drops may increase and ΔDBP responses may decline (especially in women) with age [15]. Therefore, future studies should be designed in healthy volunteers across a broad age range to establish age-dependent normative data for sympathetic indices.
3. Parasympathetic tests generally outperform sympathetic tests in balanced diagnostic accuracy. E/I ratio achieved Youden indices up to 0.84 [53], the 30:15 ratio up to 0.68 [36], and the Valsalva ratio up to 0.85 when age-adjusted thresholds were applied [31]. In contrast, ΔSBP and ΔDBP had high specificity but low-to-moderate sensitivity [27, 31, 32, 76] limiting their value as early screening tools and supporting their primary use as confirmatory measures in established cases [77].
4. No consistent sex-specific cutoffs are applied despite well-known physiological differences [9, 15, 78]. For example, although female participants often had slightly higher Valsalva ratios and slightly lower ΔDBP responses than male participants [15], no study applied separate diagnostic thresholds by sex.
5. Methodological differences in defining and conducting the tests were evident across studies. The majority of studies ensured patients rested (often ≥ 2 min) in a supine position before testing, thereby standardizing baseline HR or BP and minimizing variability due to transient physiological fluctuations, although rest intervals and baseline adjustments were not clearly defined across all studies. The incorporation of such baseline-related adjustments, proper preparation of the patient and standardization of the environment represents an important methodological refinement that could improve diagnostic precision and is supported by the consensus statement on electrodiagnostic assessment of the ANS, endorsed by the American Autonomic Society, the American Academy of Neurology, and the International Federation of Clinical Neurophysiology [78].
6. There is a marked heterogeneity in diagnostic criteria across studies: The most common approach was the classical Ewing criterion of ≥ 2/5 abnormal tests. Studies that applied age-adjusted cutoffs and balanced point assignment achieved strong results [27, 31, 36]. Studies involving diagnostic reference standard of HR-based tests usually attained adequate sensitivity without compromising specificity [28, 32, 70], as recommended by the Toronto Consensus Panel and other expert recommendations [11, 79, 80]. This variability reduces comparability between studies and complicates pooled analyses. For clinical diagnosis of definite dysautonomia the use of the classical criterion of ≥ 2/5 abnormal tests incorporating age-adjusted normative cutoffs remains the most validated and reproducible approach and aligns with international guidelines [11, 12, 81].
7. The combination of all five Ewing tests approach yielded strong diagnostic performance across disease cohorts, whereas the accuracy of single tests is more vulnerable to test selection and cutoff approach. This finding is consistent with previous literature [15, 78, 82] which has demonstrated greater diagnostic utility when the Ewing tests are interpreted in combination rather than on an individual test basis. These findings provide further justification for the routine application of the full Ewing battery—interpreted with validated diagnostic reference standard and age-corrected reference values.

Consequently, in order to achieve optimal diagnostic precision, every laboratory should apply a standardized methodology for performing the tests and establish its own age- and population-specific normative cutoffs derived from well-characterised local healthy cohorts. Such locally validated reference values ensure that physiological differences, demographic profiles, and methodological nuances are fully accounted for clinical interpretation. Until such datasets become available, the consolidated evidence from this systematic review provides a reliable reference framework ensuring that clinical and research practice is guided by the most reproducible and evidence-based criteria currently available.

Additionally, based on the consolidated methodological recommendations regarding the accurate and standardized implementation of cardiovascular autonomic testing [2, 4, 6–8, 10–12, 15, 33, 34, 78, 80, 81], the following should be considered when performing ANS studies:

- Standardized environment: quiet room, morning testing hours or at a consistent time of day to minimize diurnal variability, room temperature of 22–24 °C [11, 12]'
- Patient preparation: Avoid nicotine, caffeine, and large meals for ≥ 2 h; no strenuous exercise for ≥ 24 h; drug recording and, if possible, withholding of autonomically active medications including β-blockers, antidepressants, antipsychotics, antihypertensives [11, 12].
- Ensure a sufficient resting baseline period (for at least 20 min according to Low [84]) to ensure stabilization. Responses are then expressed as changes from this baseline [33, 34, 84].
- Ensure a sufficient resting period of at least 5 min between the different tests [12, 84].
- Data quality and documentation: Artefacts (ectopics, signal noise) are excluded using consistent thresholds [34].
- Use of special equipment:
  - ECG with R–R interval resolution ≥ 250 Hz (preferably 1000 Hz) [34].
  - Beat-to-beat BP monitor (e.g. Finapres) or validated automated cuff device [12, 34, 78, 84].
  - Handgrip dynamometer for isometric contraction [12, 34].
  - Metronome or software for paced breathing, and a mouthpiece with manometer for Valsalva manoeuvre [12].
- Use laboratory-based cutoffs. As a result of the variability of normative data across ages and sexes each laboratory is advised to obtain its own normative data for each procedure. Moreover, for the full Ewing battery, the ≥ 2 criterion of pathological tests is the most practical and reproducible method of diagnosing ANS disorder.
- Test sequence: Only five included studies [15, 23–26] reported the test order, without providing a physiological or methodological justification, and four [23–26] did not adhere to the classical Ewing sequence. It has been suggested that parasympathetic responses may be more susceptible to prior exertion or adrenergic stimulation [17, 83]. However, the available evidence is insufficient to determine whether test order influences diagnostic performance. Further research is therefore warranted to assess whether an alternative to the classic Ewing sequence might be advisable.
- Timing and repetition of tests: No standardized evidence-based guidance exists regarding optimal test timing, number of repetitions, or inter-test recovery intervals. Across included studies, substantial heterogeneity was observed in test timing and rest periods, reflecting laboratory practice rather than empirically validated protocols. This inconsistent reporting of timing-related parameters precludes meaningful assessment of their influence on autonomic responses or diagnostic accuracy, underscoring the need for targeted methodological studies to address this gap.

We therefore adopt the original Ewing sequence as the standard test order used for descriptive purposes in this review, applying up to two repetitions per test when required criteria were not met and three repetitions for the Valsalva manoeuvre with appropriate rest intervals [12, 34, 84]. On this basis, and acknowledging the absence of evidence-based guidance, the present review uses the classical Ewing flow plan [2] as a starting framework to outline a proposed protocol aimed at improving methodological consistency and comparability across future studies, rather than as a validated or prescriptive recommendation, as the present review did not evaluate whether test order influences diagnostic performance. Specifically:

- Valsalva ratio (VR): Exhale into mouthpiece to 40 mmHg for 15 s. 3 valid trials, ≥ 3 min apart; retain best [12, 33]. Rest before next test until HR/BP return to baseline [33].
- Deep breathing test: At least 5 min of supine or sitting position before test [12, 84]. Paced at 6 breaths/min (5 s inspiration, 5 s expiration) for 1 min. Repeat twice with a resting period of 3 min [84]. Rest before next test: 5 min according to Low [84] or until HR returns to baseline [12, 33].
- Isometric handgrip (ΔDBP): Determine maximal voluntary contraction (MVC) with dynamometer. Maintain 30% MVC for up to 5 min with the dominant or non-paretic hand [12]; provide verbal encouragement. Rest after test until HR/BP return to baseline [12].
- Active stand (30:15 ratio): At least 5 min of rest in supine position before standing [12]. Quick transition from supine to standing position. Outcome: RR30 / RR15 (or HR min/max at 15th and 30th beats). Can be combined with ΔSBP measurement during the same stand.
- Orthostatic BP fall: Ideally recorded during the same active stand as 30:15 ratio. Measure the standing BP fall considering the standing values at first and second minute [12]. Baseline BP: At least 5 min of rest in supine position before standing [12]. Record exact nadir time. Rest before next test until HR/BP return to baseline (supine systolic BP for values ≥ 160 mmHg and ≤ 120 mmHg should be taken into account) [12].

This protocol has a defined structure to minimize variability, maximize reproducibility, and provide a template for the generation of population-specific normative data.

### Limitations of the study

This systematic review has some limitations that need to be considered when interpreting the results.

- Heterogeneity in study populations and protocols:
  This study included diverse patient groups, healthy control groups, and age distribution, often utilizing different recruitment criteria. In addition, variation in autonomic testing protocols, operators’ expertise, and the use of different devices or measurement systems may have accounted for differences in outcomes seen between studies.
- Absence of a universal clinical reference standard.
  Although the Ewing battery remains the most widely used test in the assessment of dysautonomia, no single clinical reference standard exists that is universally accepted to diagnose autonomic neuropathy. Inevitably, this lack of consensus affects comparability across different studies and has an impact on sensitivity and specificity.
- Lack of quantitative synthesis:
  A formal meta-analysis of diagnostic accuracy could not be performed because of profound heterogeneity in Ewing test protocols, cutoff thresholds, disease populations, and definitions of autonomic dysfunction across studies. These differences precluded meaningful pooling of sensitivity and specificity estimates and rendered quantitative synthesis (e.g. forest plots or* I*^2^ statistics) inappropriate. Consequently, no formal investigation of heterogeneity or sensitivity analyses was feasible. A statistical synthesis of results was likewise not undertaken. A formal assessment of reporting bias could not be performed because no pooled effect estimates were available; moreover, incomplete and inconsistent reporting of numerical outcomes of sensitivity and specificity across studies is acknowledged as a limitation. For the same reason, a formal certainty-of-evidence evaluation (e.g. GRADE-DTA) was not possible.
- Exclusion of conference presentations and grey literature:
  Relevant studies presented only at conferences or published as abstracts in congress proceedings may have been missed; however, most high-quality research is subsequently published as full manuscripts. The same consideration applies to grey literature, the exclusion of which may have limited the capture of all available evidence.

## Conclusion

The findings of this review highlight the need for clearer, standardized methodology and more consistent application of Ewing tests in clinical practice. Future research should generate robust age-adjusted normative values and adopt uniform, fully reported testing protocols to support reliable clinical interpretation and enable future quantitative synthesis.

## Supplementary Information

Below is the link to the electronic supplementary material.

Supplementary file1 (DOCX 270 KB)

Supplementary file2 (DOCX 34 KB)

## Data Availability

All extracted data, data extraction tables, and full search strategies are provided in the Supplementary Materials accompanying this review. No statistical code was generated as no meta-analysis was performed.
