# Supplementary material for: Cutoffs, sensitivity and specificity of the Ewing battery in evaluating autonomic nervous system disorders: a systematic review
Source: Clin Auton Res. 2026 Jan 20;36(2):155–73. doi: 10.1007/s10286-025-01185-x (PMC13068710; doi:10.1007/s10286-025-01185-x)
Supplement: Supplementary file 2 — Supplementary file2 (DOCX 34 KB) [file 10286_2025_1185_MOESM2_ESM.docx]

**Table S1.** Summary of quality assessment for cross-sectional studies (n=41)

| **Author, Year** | **1** | **2** | **3** | **4** | **5** | **6** | **7** | **8** | **Total score** |  |
| --- | --- | --- | --- | --- | --- | --- | --- | --- | --- | --- |
|  | **Clearly defined inclusion criteria** | **Residents and setting described in detail** | **Exposure measured in a valid and reliable way** | **Objective, standard criteria used for condition measurement** | **Confounding factors identified** | **Strategies to deal with confounding factors stated** | **Outcomes measured in a valid and reliable way** | **Statistical analysis appropriate** |  | **ROB** |
| [Awe et al, 2022](https://pubmed.ncbi.nlm.nih.gov/?size=200&term=Awe+OO&cauthor_id=34739627) | YES | YES | ΥΕΣ | YES | YES | YES | YES | YES | 8 | LOW |
| [Syngle et al.,2016](https://pubmed.ncbi.nlm.nih.gov/?size=200&term=Syngle+V&cauthor_id=27503343) | YES | YES | YES | YES | YES | U | U | YES | 6 | LOW |
| Amir et al.,2024 | YES | YES | YES | YES | U | NO | YES | YES | 6 | LOW |
| Keller et al.,2024 | YES | YES | YES | YES | U | NO | YES | YES | 6 | LOW |
| Idiaquez et al.,2023 | YES | YES | YES | YES | YES | NO | YES | YES | 7 | LOW |
| Dhumad et al.,2021 | YES | YES | YES | YES | YES | YES | YES | YES | 8 | LOW |
| Syngle et al.,2016 | YES | YES | YES | YES | YES | U | YES | YES | 6 | LOW |
| Viswanathan et al.,2000 | YES | YES | YES | YES | U | NO | YES | YES | 6 | LOW |
| Corazza et al.,2014 | YES | YES | U | YES | U | NO | YES | YES | 5 | MODERATE |
| Syngle et al.,2015 | YES | YES | YES | YES | YES | YES | U | YES | 7 | LOW |
| Matei et al.,2013 | YES | YES | YES | YES | YES | YES | YES | YES | 8 | LOW |
| Ewing et al.,1985 | YES | YES | YES | YES | U | U | YES | YES | 6 | LOW |
| Nikolić et al.,2014 | YES | YES | YES | YES | YES | U | YES | YES | 7 | LOW |
| Lee et al.,2018 | YES | YES | YES | YES | U | NO | U | YES | 5 | MODERATE |
| Montesano et al.,2010 | YES | YES | YES | YES | U | NO | YES | YES | 6 | LOW |
| Körei et al.,2024 | YES | YES | YES | YES | YES | YES | YES | YES | 8 | LOW |
| Gunal et al.,2002 | YES | YES | YES | YES | U | NO | YES | YES | 6 | LOW |
| Agelink et al.,1998 | YES | YES | YES | YES | YES | YES | YES | YES | 8 | LOW |
| He et al., 2003 | YES | YES | YES | YES | U | NO | YES | YES | 6 | LOW |
| Palová et al.,2010 | YES | YES | YES | YES | U | NO | YES | YES | 6 | LOW |
| Tank et al.,2001 | U | YES | YES | YES | U | NO | YES | YES | 5 | MODERATE |
| Nussinovitch et al.,2011 | YES | YES | YES | YES | U | NO | YES | YES | 6 | LOW |
| Kamińska et al,2008 | YES | YES | YES | YES | U | NO | YES | YES | 6 | LOW |
| Tsiompanidis et al.,2018 | YES | YES | YES | YES | YES | YES | YES | YES | 8 | LOW |
| Xiong et al.,2014 | YES | YES | YES | YES | YES | YES | YES | YES | 8 | LOW |
| Shrivastava et al.,2023 | YES | YES | YES | YES | NO | NO | YES | YES | 6 | LOW |
| Aggarwal et al.,2017 | YES | YES | YES | YES | U | NO | YES | YES | 6 | LOW |
| Peric et al.,2011 | YES | YES | YES | YES | YES | YES | YES | YES | 8 | LOW |
| Peng et al.,2021 | YES | YES | YES | YES | U | NO | YES | YES | 6 | LOW |
| Lin et al.,2017 | YES | YES | YES | YES | U | NO | YES | YES | 6 | LOW |
| Xiong et al.,2012 | YES | YES | YES | YES | YES | YES | YES | YES | 8 | LOW |
| Brisinda et al.,2014 | YES | YES | YES | YES | U | NO | YES | YES | 6 | LOW |
| Nowicki et al.,2009 | YES | YES | YES | YES | U | NO | YES | YES | 6 | LOW |
| Xiong et al.,2013 | YES | YES | YES | YES | YES | YES | YES | YES | 8 | LOW |
| Psallas et al.,2006 | YES | YES | YES | YES | U | NO | YES | YES | 6 | LOW |
| Ducher et al.,1999 | YES | YES | YES | YES | YES | NO | YES | YES | 7 | LOW |
| Pavlovic et al.,2010 | YES | YES | YES | YES | U | NO | YES | YES | 6 | LOW |
| Mestivier et al.,1997 | YES | YES | YES | YES | U | YES | YES | YES | 7 | LOW |
| Cardone et al.,1990 | YES | YES | YES | YES | U | U | YES | YES | 6 | LOW |
| Kamińska et al,2008 | YES | YES | YES | YES | U | U | YES | YES | 6 | LOW |
| Ducher et al.,2001 | YES | YES | YES | YES | U | NO | YES | YES | 6 | LOW |

**Table S2.** Summary of quality assessment for cohort studies (n=2)

| **Author, Year** | **1** | **2** | **3** | **4** | **5** | **6** | **7** | **8** | **9** | **10** | **11** | **Total score** |  |
| --- | --- | --- | --- | --- | --- | --- | --- | --- | --- | --- | --- | --- | --- |
|  | **Similar study groups recruited from same population** | **Exposures measured similarly in assignment to groups** | **Exposure measured in a valid and reliable way** | **Confounding factors identified** | **Strategies to deal with confounding factors stated** | **Residents free of outcome at start of study** | **Outcomes measured in a valid and reliable way** | **Follow-up time reported** | **Follow-up complete. If not, reasons described** | **Strategies to address incomplete follow up utilized** | **Statistical analysis appropriate** |  | **ROB** |
| Gerritsen et al.,2000 | YES | YES | YES | YES | YES | YES | YES | N/A | N/A | N/A | YES | 8 | LOW |
| Baschieri et al.,2015 | YES | YES | YES | YES | YES | YES | YES | YES | YES | N/A | YES | 10 | LOW |

**Table S3.** Summary of quality assessment for case control studies (n=7)

| **Author, Year** | **1** | **2** | **3** | **4** | **5** | **6** | **7** | **8** | **9** | **10** | **Total score** |  |
| --- | --- | --- | --- | --- | --- | --- | --- | --- | --- | --- | --- | --- |
|  | **Case control groups are comparable** | **Cases and controls matched appropriately** | **Same criteria used for identification** | **Exposure measured in a valid and reliable way** | **Exposure measured in the same way** | **Confounding factors identified** | **Strategies to deal with confounding factors stated** | **Outcomes measured in a valid and reliable way** | **Was the exposure period of interest long enough to be meaningful** | **Statistical analysis appropriate** |  | **ROB** |
| Gujjar et al.,2023 | YES | YES | YES | YES | YES | U | U | YES | N/A | YES | 7 | LOW |
| Allan et al.,2007 | YES | YES | YES | YES | YES | U | U | YES | N/A | YES | 7 | LOW |
| Naz et al.,2021 | YES | YES | YES | YES | YES | U | U | YES | N/A | YES | 7 | LOW |
| Videira et al.,2016 | YES | YES | YES | YES | YES | U | U | YES | N/A | YES | 7 | LOW |
| Bonete et al.,2019 | YES | YES | YES | YES | YES | U | U | YES | Ν/Α | YES | 7 | LOW |
| Milovanović et al.,2010 | YES | NO | YES | YES | YES | U | NO | YES | N/A | YES | 6 | MODERATE |
| Khandelwal et al.,2023 | YES | YES | YES | YES | YES | U | U | YES | N/A | YES | 7 | LOW |

**Table S4.** Summary of quality assessment for RCTs (n=1)

Author___________ Syngle et al____________________________ Year_____2014____

|  | Yes | No | Unclear | NA |
| --- | --- | --- | --- | --- |
| 1. Was true randomization used for assignment of participants to treatment groups? | x | □ | □ | □ |
| 1. Was allocation to treatment groups concealed? | □ | □ | x | □ |
| 1. Were treatment groups similar at the baseline? | x | □ | □ | □ |
| 1. Were participants blind to treatment assignment? | x | □ | □ | □ |
| 1. Were those delivering treatment blind to treatment assignment? | x | □ | □ | □ |
| 1. Were outcomes assessors blind to treatment assignment? | x | □ | □ | □ |
| 1. Were treatment groups treated identically other than the intervention of interest? | x | □ | □ | □ |
| 1. Was follow up complete and if not, were differences between groups in terms of their follow up adequately described and analyzed? | x | □ | □ | □ |
| 1. Were participants analyzed in the groups to which they were randomized? | □ | □ | x | □ |
| 1. Were outcomes measured in the same way for treatment groups? | x | □ | □ | □ |
| 1. Were outcomes measured in a reliable way? | x | □ | □ | □ |
| 1. Was appropriate statistical analysis used? | x | □ | □ | □ |
| 1. Was the trial design appropriate, and any deviations from the standard RCT design (individual randomization, parallel groups) accounted for in the conduct and analysis of the trial? | x | □ | □ | □ |

Overall appraisal: Include x Exclude □ Seek further info

_Score:11/13______________ROB: LOW______________________________________________________

Footnote:

1. If the answer is yes, the question is assigned a score of 1. If the answer is no, unclear, or not applicable, it is assigned a score of 0. Studies categorized as low, moderate, or high risk of bias based on the total score. For randomized controlled trials: low ≥10/13, moderate 7–9/13, high ≤6/13; cohort studies: low ≥8/11, moderate 6–7/11, high ≤5/11; case–control studies: low ≥7/10, moderate 5–6/10, high ≤4/10; analytical cross-sectional studies: low ≥6/8, moderate 4–5/8, high ≤3/8).A score of 4 to 6 indicates moderate quality, whereas as score of 7 or more indicates high quality.
